# Theory of Dreams

**Published:** 1879-10

**Authors:** 


ANOTHER THEORY CONCERNING DREAMS.—A novel and interesting theory as to the nature of dreams was lately propounded before the Royal Medical Institution, by Prof. Humphrey, of Cambridge, England, defining dreams as not a normal accompaniment of sleep, but rather a result of the abnormal or imperfect condition of the organ of mental action. In the natural state, he says, we should pass from wakefulness to complete unconsciousness, and *vice versa*, almost instantaneously, and this is the case with many persons ; more frequently, however, the transition is protracted. and stages are observed in which the sleep is but partial In this case, according to Prof. Humphrey, the cerebral organ being in an imperfect state, its action is imperfect, and the first effect of the lessening of its vital vigor is a loss of the highest form of mental power—the control over the mental operations; and, in this condition, the thoughts ramble unchecked, chase one another confusedly over the mental field, and give rise to all sorts of incongruities of the imagination.

